# Food Label Reading and Cardiovascular Health Profiles Across Adulthood: Findings from the Look-Up 8+ Study

**DOI:** 10.3390/nu18213463

**Published:** 2026-10-22

**Authors:** Anna Maria Martone, Elena Levati, Riccardo Calvani, Matteo Tosato, Vincenzo Galluzzo, Francesca Ciciarello, Sara Salini, Stefano Cacciatore, Emanuele Marzetti, Francesco Landi

**Affiliations:** 1 Center for Aging and Longevity Medicine (CEMI), Fondazione Policlinico Universitario “Agostino Gemelli” IRCCS, Largo Agostino Gemelli 8, 00168 Rome, Italy; 2 Department of Geriatrics, Orthopedics and Rheumatology, Università Cattolica del Sacro Cuore, Largo Francesco Vito 1, 00168 Rome, Italy; 3 Department of Physiology and Aging, College of Medicine, University of Florida, Gainesville, FL 32611, USA

**Keywords:** food label, cardiovascular, dietary patterns

## Abstract

**Background**: Unhealthy dietary patterns are a major risk factor for death and functional disability. Food labeling provides key nutritional information and is associated with healthier dietary patterns. This study aims to investigate whether attention to food labels is associated with dietary patterns, BMI, and Cardiovascular 8 metrics scores. **Methods**: We analyzed data from the Look-Up 8+ project, including adults aged 18 years or older from several Italian regions. Participants underwent a comprehensive lifestyle assessment, including dietary habits, cholesterol and glucose measurements, and anthropometric evaluations. Descriptive, comparative, and inferential statistical analyses were used to investigate the association between attention to food labels and dietary patterns, BMI, and Cardiovascular Health scores. **Results**: Among 7401 participants (mean age 54.4 ± 15.0 years, with 55.5% women), those who reported reading food labels exhibited a higher prevalence of regular physical activity and adherence to a healthy diet. In contrast, participants who did not read food labels had a higher body mass index. Both men and women who reported greater attention to food composition had a higher Life’s Essential 8 score. **Conclusions**: These results suggest that attention to food labels is associated with more favorable lifestyle choices and improved cardiovascular health profiles.

## 1. Introduction

Unhealthy dietary patterns are a major risk factor for death and functional disability, due to their relationship with non-communicable diseases (NCDs), particularly hypertension, obesity, and type 2 diabetes [1]. In 2019, an estimated 7.9 million deaths worldwide were attributable to dietary risk factors, with high sodium intake and low whole-grain intake among the leading contributors [2]. The role of nutritional factors in cardiovascular health is well-established. Saturated and trans fats, refined carbohydrates, high sodium intake and ultra-processed foods contribute to increased cardiovascular disease risk. Conversely, diets rich in fiber, plant-based proteins, potassium and omega-3 acids are associated with lower morbidity and mortality [3,4,5].

Despite widespread public health recommendations promoting increased adherence to healthy dietary guidelines remains suboptimal in many populations worldwide. Providing clear nutritional information about products may empower consumers to make informed choices about food and drink options and encourage companies to enhance transparency and improve the nutritional profile in food preparation.

Food labeling provides key nutritional information, typically including calories, macronutrients, and selected micronutrients, in a visible format [6], with the aim of improving transparency and empowering consumers at the point of purchase offering a way to impact in public health and reduce incidence of NCDs [7,8].

Evidence indicates that reading nutrition labels is associated with healthier dietary patterns, including higher intake of fruits, vegetables, and fiber, and lower consumption of saturated fats, added sugars, and sodium [9]. However, the relationship varies depending on which label components consumers use—for example, only those who specifically check sugar information tend to consume less added sugar [9]. Countries implementing nutrition labels have documented declines in sodium intake, added sugars, and unhealthy fats, supporting mandatory labelling policies. Based on these international experiences, particularly in Latin America, the World Health Organization (WHO) and Food and Drug Administration (FDA) endorse front-of-package labels as supplementary nutrition information to support healthier dietary habits [6,10].

An observational Italian study have also reported inverse associations between food label reading and body mass index (BMI), as well as lower prevalence of obesity and metabolic risk factors [11].

In recent years, composite cardiovascular health scores—such as the American Heart Association’s Life’s Essential 8 metrics [12]—have gained increasing attention as integrative tools to assess cardiovascular health status in the general population. These metrics encompass behavioral and biological factors, including diet, physical activity, body weight, blood pressure, glucose, and lipid levels, providing a holistic framework for cardiovascular prevention. However, data examining the relationship between food label literacy and global cardiovascular health scores in real-world populations remain scarce.

In this context, the Longevity Check-up 8+ project [13] study offers a unique opportunity to explore these associations in a large, community-based Italian population. This study aims to investigate the association between attention to food labels and dietary patterns, BMI, and Cardiovascular 8 metrics scores. Understanding the role of food label literacy in shaping dietary choices and cardiovascular health may inform future public health strategies aimed at improving nutritional awareness and preventing cardiometabolic diseases.

## 2. Materials and Methods

### 2.1. Study Design and Sample Selection

This present study analysed data derived from the Longevity Check-up (Look-Up) 8+ project, a nationwide observational initiative coordinated by Università Cattolica del Sacro Cuore in partnership with Fondazione Policlinico Universitario “Agostino Gemelli” IRCCS (Rome, Italy). The primary objective of the Lookup 8+ project was to promote the adoption of healthy lifestyles within the general population, assessing cardiovascular risk factors and its determinants in the adult population. The study included individuals aged 18 years or older who participated in health evaluations between 1 January 2015, and 31 December 2025. Participants were enrolled through voluntary walk-in screening campaigns conducted in public venues (such as city squares, shopping malls, and cultural or community events) across different regions of Italy. Exclusion criteria comprised self-reported pregnancy, refusal to undergo capillary blood sampling for the assessment of cholesterol and glucose levels, and incapacity to provide written informed consent. The Lookup 8+ protocol was approved by the Ethics Committee of Università Cattolica del Sacro Cuore (Protocol No. A.1220/CE/2011; approval date: 3 March 2014), and all participants provided written informed consent prior to enrollment. Additional details regarding the study design, aims, and assessment procedures have been previously reported [14,15].

### 2.2. Data Collection

Participants underwent a comprehensive assessment, which included a lifestyle questionnaire, cholesterol and glucose measurements, and evaluations of anthropometric parameters. Lifestyle habits were collected through a structured interviewer-administered questionnaire. Smoking status was classified as current, former, or never smoker. Dietary habits were evaluated using a simplified food frequency questionnaire covering the major food groups [16], with portion sizes estimated according to standard Italian reference servings [17]. Based on self-reported intake of main food items, a healthy diet was defined according to fruit and vegetable consumption, with three or more daily portions categorized as indicative of a healthy diet; this is a simplified indicator based exclusively on fruit and vegetable intake, and does not capture overall diet quality, which was assessed separately with the MEDI-LITE score. Adherence to the Mediterranean diet was additionally assessed using the validated MEDI-LITE score [18]. Body weight was measured using a calibrated analog scale, and height was assessed with a portable stadiometer; body mass index (BMI) was calculated as weight in kilograms divided by height in meters squared (kg/m^2^). Physical activity was defined as participation in any form of exercise for at least 30 min on two or more days per week during the preceding year. Although less stringent than current World Health Organization recommendations, this definition was adopted to ensure feasibility and standardization across heterogeneous field settings [19]. Information on the type of physical activity was also collected. For analytical purposes, participants were categorized as physically active or inactive. Capillary blood samples were obtained by fingertip puncture using sterile, single-use devices. Participants reported whether they had fasted for at least two hours prior to sampling, allowing classification of measurements as fasting or non-fasting. Total cholesterol and blood glucose levels were assessed using portable point-of-care analyzers with disposable test strips. Hypercholesterolemia was defined as total cholesterol >200 mg/dL or ongoing lipid-lowering therapy. Diabetes was defined as fasting blood glucose ≥126 mg/dL, random glucose ≥200 mg/dL, or a self-reported physician diagnosis. Blood pressure was measured in the seated position using an automated sphygmomanometer, according to standardized procedures [20]. Hypertension was defined as systolic pressure ≥140 mmHg, diastolic pressure ≥90 mmHg, or current antihypertensive treatment. The 8 cardiovascular health score was calculated as a composite measure encompassing eight key health behaviors and risk factors: diet, physical activity, smoking, sleep, BMI, blood pressure, total blood cholesterol, and blood glucose. Each component was assigned a scaled score ranging from 0 to 100, with higher values indicating optimal cardiovascular health, and the final score was derived as the average of these components.

### 2.3. Food Label Reading Status

Participants’ engagement with food label information was assessed using a structured, interviewer-administered question exploring both attention to and interest in nutritional and compositional information when purchasing food products. Specifically, participants were asked to report their usual behavior regarding food label reading, with response options including: “I always read labels carefully,” “I read them sometimes,” “I never read them,” and “I am not interested.” This variable was conceptualized as an indicator of nutrition-related awareness and health-oriented consumer behavior. For analytical purposes, participants who reported reading labels “always” were classified as consistent readers, whereas those reporting “sometimes,” “never,” or “not interested” were classified as less frequent/non-readers. This binary classification was adopted partly because the “never” (*n* = 172) and “not interested” (*n* = 50) categories were too small to be analyzed separately.”

### 2.4. Participants

Between 1 January 2025 and 31 December 2025, a total of 7492 adults aged ≥18 years participated in the Look-Up project and completed the assessment. The present analysis was restricted to data collected during this period, corresponding to the years in which information on food label reading was systematically assessed. Participants with missing data on key variables of interest were excluded. Specifically, 38 individuals were excluded due to missing blood pressure measurements, 25 due to missing total cholesterol values, and 28 due to missing blood glucose data. After these exclusions, the final analytical sample consisted 7401 participants, of whom 3291 were men and 4110 were women. No significant differences in demographic characteristics or cardiovascular health metrics were observed between included and excluded participants.

### 2.5. Statistical Analysis

Participant characteristics were described overall and stratified by sex and food label reading status. Descriptive statistics were used to summarize the data. The distribution of continuous variables was assessed for normality using the Kolmogorov–Smirnov test. Continuous variables are presented as mean ± standard deviation (SD), while categorical variables are expressed as absolute frequencies and percentages. Between-group comparisons for categorical variables were performed using Fisher’s exact test. For continuous variables, one-way analysis of variance (ANOVA) or the Kruskal–Wallis test was applied, as appropriate, based on data distribution. Logistic regression models were used to estimate odds ratios (ORs) and 95% confidence intervals (CIs) for the association between food label reading (consistent readers vs. less frequent/non-readers) and higher cardiovascular risk (defined as Life’s Essential 8 score below 40 points), a healthy diet, and normal BMI (<25 kg/m^2^). Models were run unadjusted and adjusted for age, gender, and education, i.e., the variables that differed significantly between food label consistent readers and less frequent/non-readers in the univariate analyses. A two-sided *p*-value <0.05 was considered statistically significant. All statistical analyses were conducted using SPSS software, version 26.0 (IBM Corp., Armonk, NY, USA). In this manuscript, AI-assisted technologies (ChatGPT v. 5.1.) was used to improve readability and language of the work but not to producing scientific insights, analyzing, and interpreting data, or drawing scientific conclusions.

## 3. Results

Among 7401 participants, mean age was 54.4 ± 15.0 years; and 55.5% were women. Baseline characteristics of participants, stratified by sex and food label reading, were reported in Table 1. Overall, women were more likely to report reading food labels compared to men (74.5% vs. 65.6%, *p* < 0.001). In men, mean age was 54.6 ± 14.6 years among food label consistent readers and 55.7 ± 15.4 years among less frequent/non-readers (*p* = 0.05), whereas in women consistent readers were older than less frequent/non-readers (54.4 ± 14.6 vs. 52.7 ± 16.4 years, *p* < 0.001). In both men and women, a higher educational level was observed among individuals who reported reading food labels compared to those who did not.

Regarding the cardiovascular health metrics, participants who reported reading food labels showed a higher prevalence of regular physical activity (defined as physical exercise at least twice a week) and adherence to a healthy diet (defined as consumption of at least three portions of fruit and/or vegetables per day). This pattern was consistent in both sexes. Among men, regular physical activity was reported in 71.6% of those who read food labels compared to 62.8% of those who did not (*p* < 0.001), while among women the corresponding proportions were 67.1% versus 56.4% (*p* < 0.001). Similarly, adherence to a healthy diet was higher among label readers. In men, 58.6% of those who read food labels reported a healthy diet compared to 42.2% of those who did not (*p* < 0.001), while in women the proportions were 70.4% versus 54.6%, respectively (*p* < 0.001). Body mass index (BMI) was higher in participants who did not read food labels, both in men and women. Among men, BMI was 25.3 kg/m^2^ in those who read labels versus 25.9 kg/m^2^ in those who did not (*p* < 0.001), while among women the corresponding values were 23.4 kg/m^2^ versus 24.1 kg/m^2^ (*p* < 0.001). Smoking was less frequent among food label consistent readers, both in men (17.5% vs. 22.3%, *p* < 0.001) and in women (16.9% vs. 19.3%, *p* = 0.04). No significant differences were observed in the prevalence of hypertension or in total cholesterol levels according to food label reading status, whereas diabetes and sleep disorders were more prevalent among men who did not read food labels compared to those who did (diabetes: 12.1% vs. 10.1%, *p* = 0.04; sleep disorders: 31.0% vs. 26.7%, *p* = 0.01), with no differences in women. Finally, the overall cardiovascular health score (Life’s Essential 8) was higher in both men (66.7 ± 12.8 vs. 62.2 ± 12.8, *p* < 0.001) and women (67.6 ± 12.9 vs. 64.4 ± 13.2, *p* < 0.001) who reported greater attention to food composition, as reflected by reading food labels always.

In logistic regression analyses (Table 2), food label reading was associated with lower odds of higher cardiovascular risk (unadjusted OR 0.58, 95% CI 0.51–0.65; adjusted OR 0.63, 95% CI 0.54–0.83), and with higher odds of a healthy diet (unadjusted OR 2.04, 95% CI 1.84–2.24; adjusted OR 2.07, 95% CI 1.82–2.35) and of normal BMI (unadjusted OR 1.49, 95% CI 1.32–1.62; adjusted OR 1.31, 95% CI 1.15–1.51). These associations remained statistically significant after adjustment for age, gender, and education. Among the covariates, age (OR 1.02 per year, 95% CI 1.01–1.03), male gender (OR 1.54, 95% CI 1.33–1.77), and education (OR 0.95 per year, 95% CI 0.94–0.97) were independently associated with higher cardiovascular risk, whereas male gender was associated with lower odds of a healthy diet (OR 0.55, 95% CI 0.49–0.63) and of normal BMI (OR 0.41, 95% CI 0.36–0.46).

**Figure 1:**
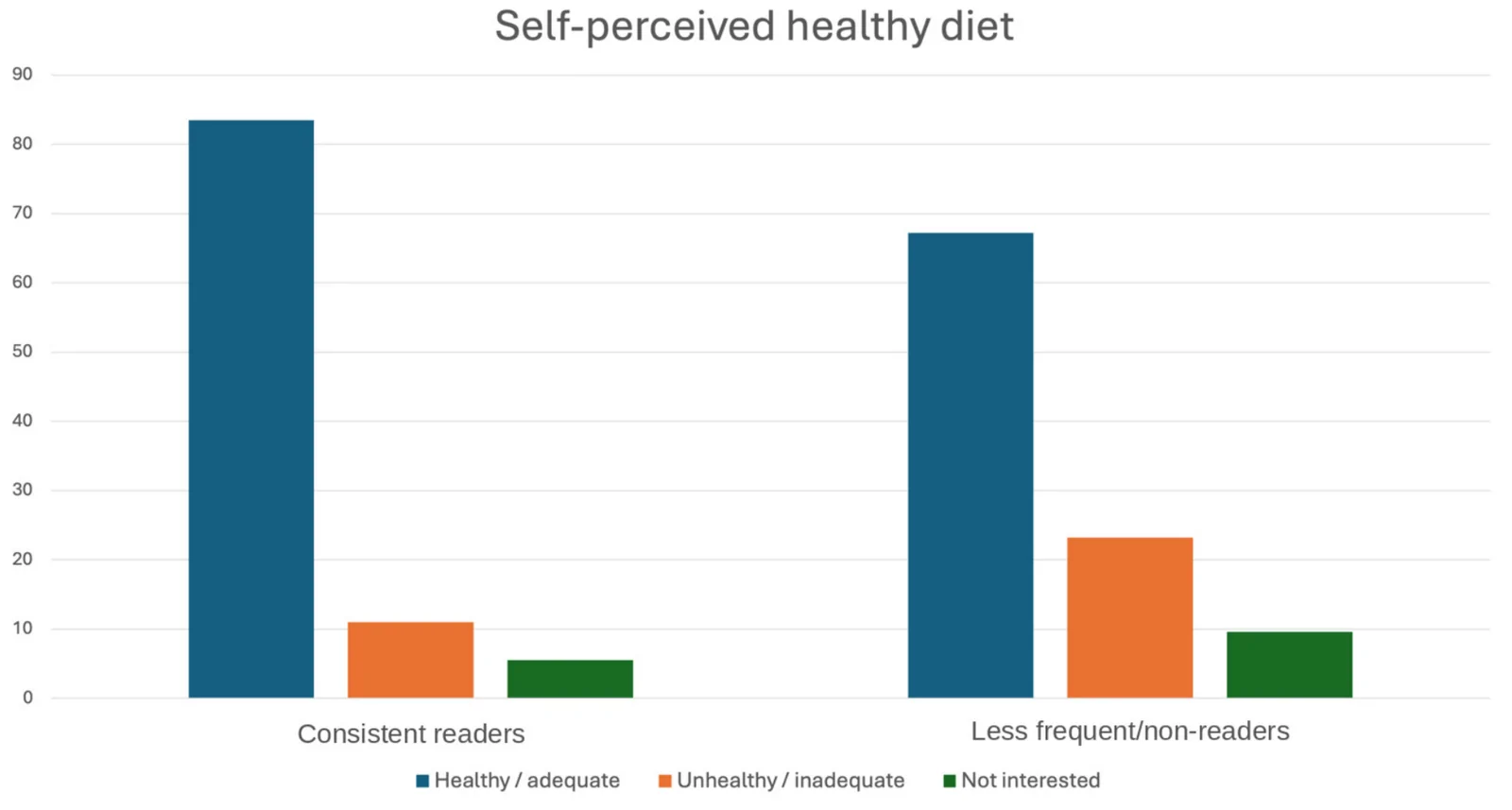
Prevalence of self-perceived healthy diet according to food label reading status.

Figure 1 illustrates the distribution of study participants according to food label reading behavior and self-perceived dietary habits. Notably, even among individuals who did not read food labels, nearly 70% perceived their diet as healthy, while more than 20% reported that their diet was not healthy. In addition, in both groups, approximately 10% of participants reported no interest in the healthiness of their diet.

**Figure 2:**
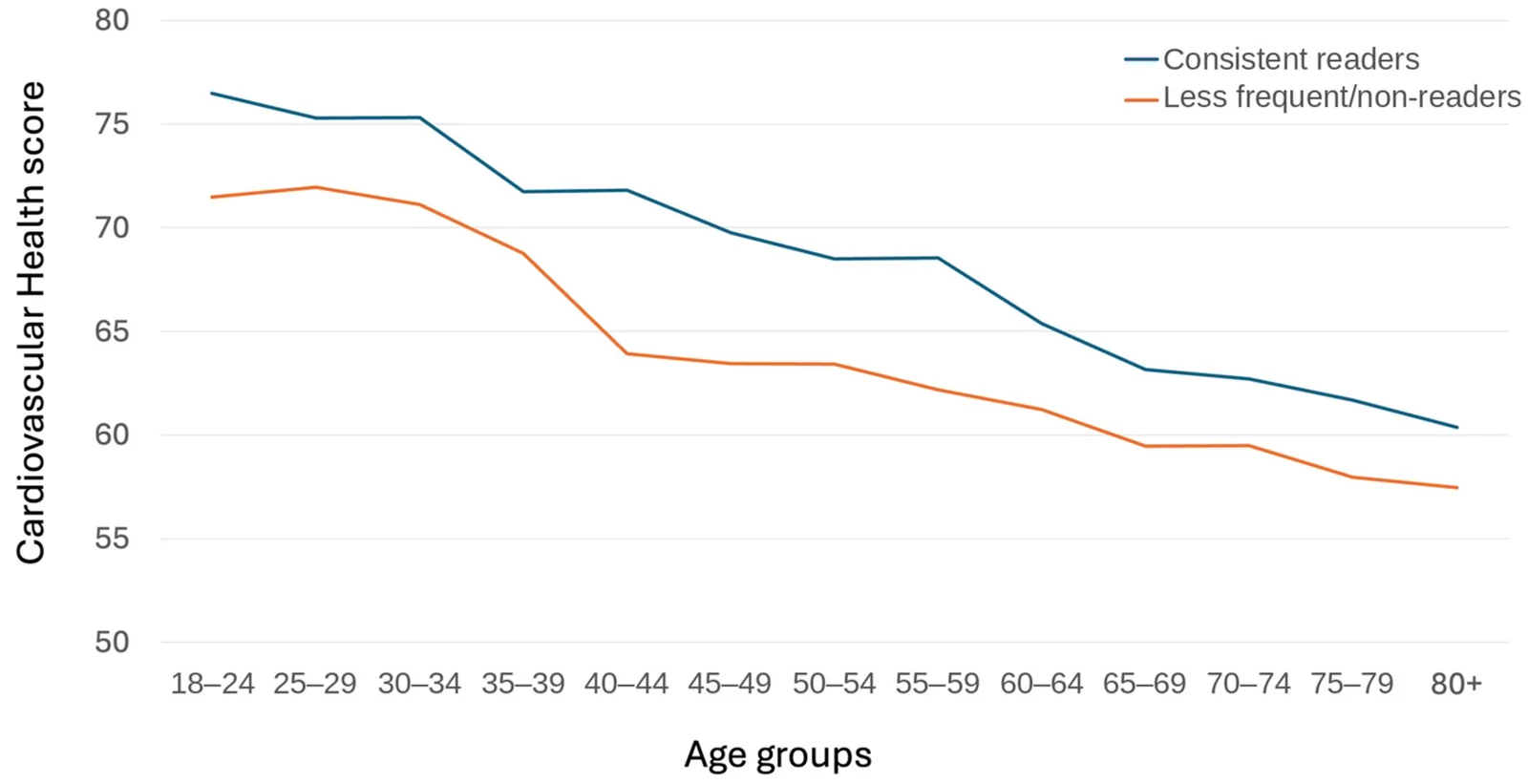
Cardiovascular Health (Life’s Essential 8) score according to food label reading status across age groups.

**Figure 3:**
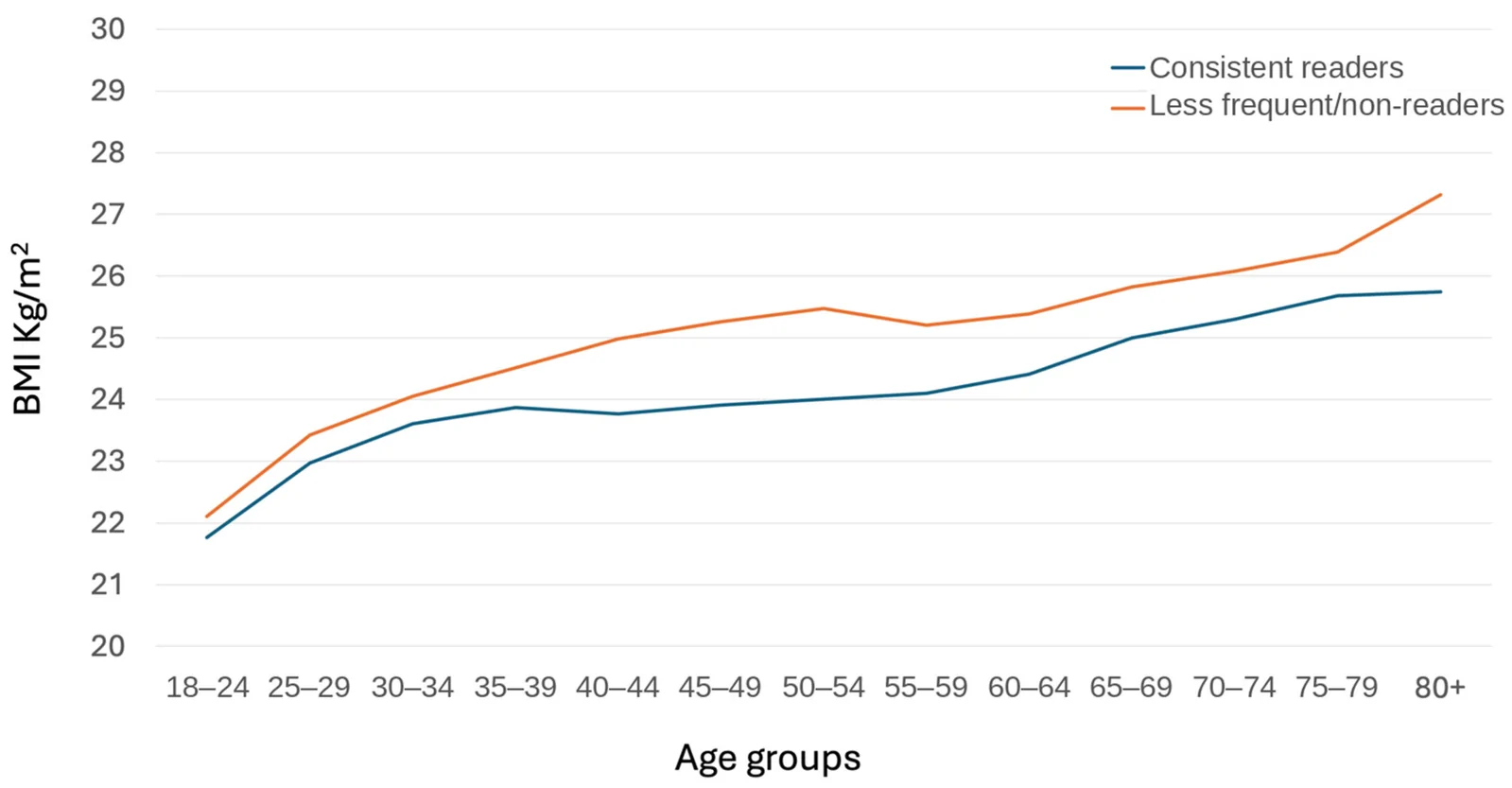
Body mass index according to food label reading status across age groups.

**Figure 4:**
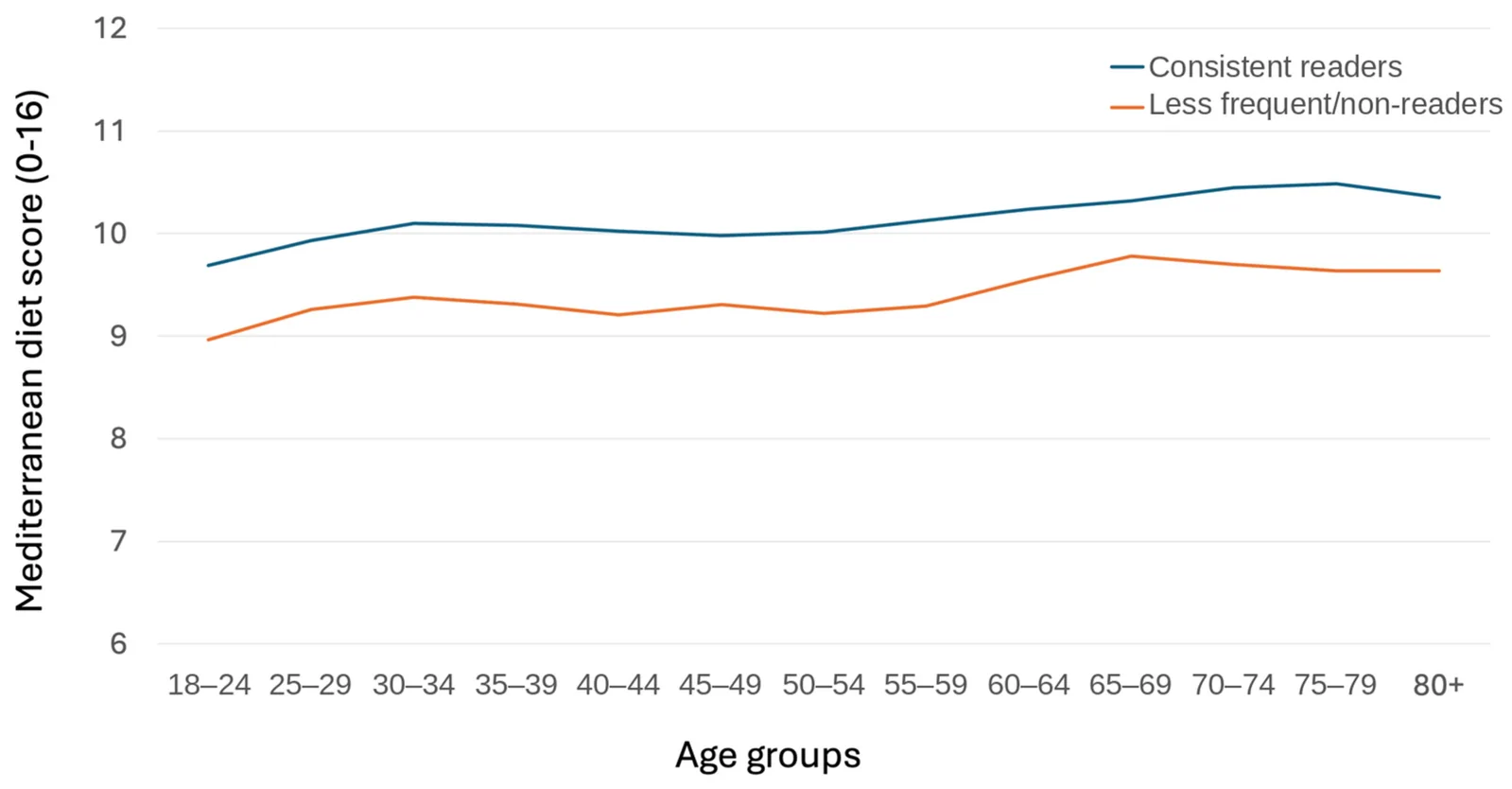
Adherence to Mediterranean diet according to food label reading status across age groups.

Figure 2, Figure 3 and Figure 4 further highlight the consistent association between food label reading and key indicators of cardiovascular health across the adult lifespan (18 years to ≥80 years). As shown in Figure 2, participants who reported reading food labels exhibited markedly higher Life’s Essential 8 scores at all ages, with a difference of at least 10 points compared to less frequent/non-readers. This gap was particularly pronounced during midlife. Figure 3 shows that BMI was consistently higher among food label less frequent/non-readers across all age groups. The difference was most evident between 40 and 60 years of age and remained clearly detectable even in older participants. Similarly, Figure 4 shows that adherence to the Mediterranean diet was systematically higher among food label consistent readers, who scored approximately one point higher on the MEDI-LITE scale at all ages. Taken together, these findings indicate that attention to food labels is consistently associated with more favorable lifestyle behaviors and cardiovascular health profiles throughout adulthood, with particularly relevant differences emerging during midlife.

## 4. Discussion

In this large cross-sectional study conducted in a real-world Italian population, we observed that individuals who reported reading food labels were more likely to exhibit healthier lifestyle behaviors and more favorable cardiovascular health profiles. Specifically, food label consistent readers showed higher levels of physical activity, greater adherence to a healthy diet and the Mediterranean dietary pattern, lower BMI, and higher overall Life’s Essential 8 scores compared to less frequent/non-readers. These associations were consistent across sexes and across a wide age range, from early adulthood to older age. The associations with cardiovascular risk, healthy diet, and BMI remained statistically significant after adjustment for age, gender, and education.

Our findings are in line with the previous literature suggesting that food label use is associated with healthier dietary choices and improved cardiometabolic profiles [21,22]. Individuals who engage with nutritional information may be more aware of the composition of the foods they consume and therefore more likely to make health-conscious decisions. Notably, in our study, the association between food label reading and healthier behaviors extended beyond diet alone, encompassing physical activity and overall cardiovascular health metrics [23,24]. This supports the hypothesis that food label reading may represent a broader marker of health-oriented behavior and lifestyle awareness, rather than an isolated dietary habit.

A particularly relevant finding is the consistent difference in Life’s Essential 8 scores between consistent readers and ess frequent/non-readers across all age groups, with a gap of at least 10 points. This difference was most pronounced during midlife, a critical period for the development and progression of cardiovascular risk [25]. This pattern is consistent with more favorable cardiovascular health profiles among food label readers across age groups. Similarly, the observed differences in BMI across all age groups, especially between 40 and 60 years, are in line with an association between food-related decision-making and body weight.

Another noteworthy result is the higher adherence to the Mediterranean diet among food label readers, consistently observed across all age groups. Given the well-established protective role of the Mediterranean diet in cardiovascular prevention [26,27], this finding further supports the relevance of food label literacy as a potential lever for improving diet quality at the population level.

Despite these favorable associations, we also observed a substantial mismatch between perceived and actual dietary behaviors. A large proportion of participants who did not read food labels still perceived their diet as healthy, highlighting a potential gap in nutritional awareness. This misperception may limit the effectiveness of public health strategies if individuals are not able to accurately assess the quality of their own diet [28,29]. From a behavioral epidemiology perspective, this finding underscores the importance of not only providing nutritional information but also improving individuals’ ability to interpret and apply it in daily choices [30].

Interestingly, no significant differences were observed in the prevalence of hypertension, in total cholesterol levels, or in most metabolic conditions according to food label reading status, except for diabetes and sleep disorders in men. This may reflect the cross-sectional nature of the study, where lifestyle behaviors may precede measurable clinical differences, or the relatively early stage of risk factor development in a portion of the population [31]. It also suggests that food label reading may be more strongly associated with upstream behavioral determinants rather than established clinical conditions.

The present study has several strengths. It is based on a large sample size including more than 7400 participants, enhancing statistical power and the robustness of the findings. The study population was recruited in real-life settings across multiple Italian regions, increasing the heterogeneity and generalizability of the results to the adult population. Furthermore, the availability of standardized measures of lifestyle behaviors, anthropometric parameters, and multiple cardiovascular health metrics allowed for a comprehensive assessment of cardiovascular health using an integrated framework such as Life’s Essential 8. However, some limitations should be acknowledged. First, the cross-sectional design precludes any inference on causality or the directionality of the observed associations. It is not possible to determine whether food label reading leads to healthier behaviors or whether individuals with healthier lifestyles are more likely to read food labels. Second, food label reading behavior and dietary habits were self-reported, which may be subject to recall bias and social desirability bias. Third, the assessment of diet was based on simplified measures, which, although suitable for field-based data collection, may not capture the full complexity of dietary patterns. In addition, combining “sometimes,” “never,” and “not interested” into a single comparison group may obscure heterogeneity in food-label engagement; future studies should evaluate the frequency of label reading in a more graded manner. Finally although the regression analyses were adjusted for age, gender, and education, unmeasured confounding factors, such as health literacy, socioeconomic status beyond education, or psychological determinants, may have influenced the observed associations.

## 5. Conclusions

Attention to food label information is consistently associated with healthier lifestyle behaviors and more favorable cardiovascular health profiles across the adult lifespan. These findings suggest that food label literacy may represent an important, modifiable target for public health interventions aimed at improving diet quality and preventing cardiometabolic diseases. Future longitudinal studies are needed to clarify the causal pathways underlying these associations and to evaluate the effectiveness of strategies designed to enhance the use and understanding of food labels in the general population.

## Figures and Tables

**Table 1:** Characteristics of the study population according to sex and food label reading.

Characteristics	Total Sample (*n* = 7401)	MALE (*n* = 3291)		*p*	FEMALE (*n* = 4110)		*p*
		Food Label Consistent Readers (*n* = 2158)	Food Label Less Frequent/Non-Readers (*n* = 1133)		Food Label Consistent Readers (*n* = 3061)	Food Label Less Frequent/Non-Readers (*n* = 1049)	
**Age, years**	54.4 ± 15.0	54.6 ± 14.6	55.7 ± 15.4	0.05	54.4 ± 14.6	52.7 ± 16.4	<0.001
**Education, years**	14.9 ± 4.2	15.3 ± 4.1	14.4 ± 4.4	<0.001	15.0 ± 4.1	14.1 ± 4.7	<0.001
**Cardiovascular Health Metrics**							
**Smoking**	1347 (18.2)	376 (17.5)	252 (22.3)	<0.001	517 (16.9)	202 (19.3)	0.04
**Regular physical activity**	4881 (66.4)	1538 (71.3)	706 (62.8)	<0.001	2052 (67.1)	585 (56.4)	<0.001
**Healthy diet**	4457 (60.2)	1260 (58.6)	476 (42.2)	<0.001	2150 (70.4)	571 (54.6)	<0.001
**Body Mass Index (kg/m^2^)**	24.5 ± 3.9	25.3 ± 3.4	25.9 ± 3.6	<0.001	23.4 ± 3.9	24.1 ± 4.3	<0.001
**Hypertension**	3871 (52.3)	1371 (63.9)	738 (65.7)	0.18	1334 (43.8)	428 (41.4)	0.09
**Diabetes**	668 (9.2)	212 (10.1)	134 (12.1)	0.04	234 (7.8)	88 (8.5)	0.24
**Cholesterol (mg/dL)**	191.3 ± 39.8	179.2 ± 35.5	178.4 ± 36.9	0.54	199.3 ± 38.5	197.2 ± 36.4	0.33
**Sleep disorder**	2382 (32.3)	574 (26.7)	349 (31.0)	0.01	1073 (35.2)	386 (37.1)	0.15
**Total 8 health metric (0–100)**	66.0 ± 13.1	66.7 ± 12.8	62.2 ± 12.8	<0.001	67.6 ± 12.9	64.4 ± 13.2	<0.001

**Table 2:** Unadjusted and adjusted odds ratios (95% CI) for the association of food label reading with higher cardiovascular risk, healthy diet, and normal BMI.

Characteristics	Higher Cardiovascular Risk		Healthy Diet		Normal BMI (<25 kg/m^2^)	
	Unadjusted OR (95% CI)	Adjusted OR (95% CI)	Unadjusted OR (95% CI)	Adjusted OR (95% CI)	Unadjusted OR (95% CI)	Adjusted OR (95% CI)
Food Label Reading	0.58 (0.51–0.65)	0.63 (0.54–0.83)	2.04 (1.84–2.24)	2.07 (1.82–2.35)	1.49 (1.32–1.62)	1.31 (1.15–1.51)
Age (years)	-	1.02 (1.01–1.03)	-	1.01 (1.00–1.02)	-	0.98 (0.97–0.99)
Gender (male)	-	1.54 (1.33–1.77)	-	0.55 (0.49–0.63)	-	0.41 (0.36–0.46)
Education (years)	-	0.95 (0.94–0.97)	-	1.01 (0.99–1.02)	-	1.04 (1.00–1.06)

Adjusted models include age (per year), gender (male vs. female), and education (per year). BMI = body mass index; CI = confidence interval; OR = odds ratio.

## Data Availability

The data presented in this study are available on request from the corresponding author due to privacyreasons.
